# Biocompatible Peritoneal Dialysis Fluids: Clinical Outcomes

**DOI:** 10.1155/2012/812609

**Published:** 2012-11-28

**Authors:** Yeoungjee Cho, Sunil V. Badve, Carmel M. Hawley, Kathryn Wiggins, David W. Johnson

**Affiliations:** ^1^Department of Nephrology, Princess Alexandra Hospital, University of Queensland, Level 2, ARTS Building, 199 Ipswich Road, Woolloongabba, Brisbane QLD 4102, Australia; ^2^Department of Nephrology, Royal Melbourne Hospital, Melbourne, VIC 3050, Australia

## Abstract

Peritoneal dialysis (PD) is a preferred home dialysis modality and has a number of added advantages including improved initial patient survival and cost effectiveness over haemodialysis. Despite these benefits, uptake of PD remains relatively low, especially in developed countries. Wider implementation of PD is compromised by higher technique failure from infections (e.g., PD peritonitis) and ultrafiltration failure. These are inevitable consequences of peritoneal injury, which is thought to result primarily from continuous exposure to PD fluids that are characterised by their “unphysiologic” composition. In order to overcome these barriers, a number of more biocompatible PD fluids, with neutral pH, low glucose degradation product content, and bicarbonate buffer have been manufactured over the past two decades. Several preclinical studies have demonstrated their benefit in terms of improvement in host cell defence, peritoneal membrane integrity, and cytokine profile. This paper aims to review randomised controlled trials assessing the use of biocompatible PD fluids and their effect on clinical outcomes.

## 1. Introduction 

Peritoneal dialysis (PD) is a well-established form of home-based renal replacement therapy to treat patients with end-stage kidney disease (ESKD). PD is associated with better preservation of residual renal function, initial survival advantage, reduced erythropoietic stimulatory agent requirements, and preservation of vascular access sites when compared to haemodialysis [1–3]. However, time on PD remains dismal with a 5-year technique survival in diabetic ESKD patients of only 10% in Australia [4]. Infections, predominantly PD peritonitis (25%) and peritoneal membrane failure manifesting as inadequate ultrafiltration or solute clearance (16%), are leading contributors to poor technique survival [4]. Furthermore, PD peritonitis leads to significantly increased risk of mortality [5]. 

### 1.1. Problems Associated with Conventional PD Fluids

Use of conventional PD fluids, characterised by acidic pH (5.0–5.8), high lactate concentrations (30–40 mmol/L), high osmolality (320–520 mOsm/kg), high glucose concentrations (75.5 to 214 mmol/L), and contamination by glucose degradation products (GDPs), may contribute to these adverse outcomes as demonstrated in *in vitro* and animal studies [6–9]. These “unphysiologic” characteristics of PD fluids have been associated with significant loss of peritoneal mesothelial cell viability and function, compromised peritoneal immune system, and promotion of fibrosis [6, 8–11]. Morphologic changes with continuous use of these fluids affect both the interstitial and vascular compartments of the dialysed peritoneal membrane. These include increased thickness of submesothelial compact collagenous zone and vasculopathy characterised by subendothelial hyalinization, with luminal narrowing or obliteration [12, 13]. Beyond their adverse local effects, the contents of these fluids have systemic implications, which include infusion pain [14], nephrotoxicity [15], and atherosclerosis via advanced glycation end products (AGE) promoted by GDP [16] (Table 1). 

### 1.2. An “Ideal” Biocompatible PD Fluid

An “ideal” biocompatible PD fluid should be “physiologic” to avoid these undesirable effects. It should be of neutral pH and should lack lactate buffer and GDP, with the use of nonglucose substance as an osmolar agent. This has been the holy grail in the PD community to develop a PD fluid that satisfies all of the above criteria with an ultimate goal to improve patient outcome. 

### 1.3. Currently Available Biocompatible PD Fluids

Over the past two decades, the PD fluids that are more “biocompatible” have been developed (Table 2). Minimisation of GDP formation has been achieved through the development of the multicompartment bag system which allows for heat sterilisation and storage to occur at a low pH [22] and the use of bicarbonate buffer system to lower exposure to lactate. A number of *in vitro* and *ex vivo* studies have demonstrated improvement in cellular function, in particular in the host immune system and an increase in markers of membrane integrity [9, 10, 23, 24]. Animal studies have shown improvement in ultrafiltration capacity, lower vascular endothelial growth factor (VEGF) expression, vascular density, AGE accumulation, and fibrosis with its use [20, 25]. Superior patient survival, reduction in peritonitis and exit site infection rates, and improvement in level of inflammatory markers have been reported in a number of observational studies [23, 26–28]. The aim of this article is to review the impact of these biocompatible PD fluids on clinical outcomes, based on the currently available published randomised controlled trials (RCT). 

## 2. Residual Renal Function 

Residual renal function (RRF) is a powerful prognostic indicator in patients with ESKD [29]. RRF is often reported in various forms, such as renal creatinine clearance, glomerular filtration rate (GFR), or urine volume. Eighteen RCTs described the impact of low-GDP PD fluid use on RRF (Table 3) [30–46]. Of those, improvement was seen in six studies [34, 36, 37, 42–44], and no significant difference was reported in others [30–33, 35, 38–41, 45–47]. No study has shown adverse outcome. A number of these studies were limited by single-centre setting [31, 32, 38, 40], crossover design [35, 38, 42, 43, 48], large drop-out rate (greater than 20%) [30–34, 36, 39, 42, 45], and inclusion of prevalent patients [31, 34, 35, 38, 39, 41–43, 47]. None of the single-centre studies showed difference in RRF between groups. 

The balANZ trial [44], to date, is the largest (*n* = 185), investigator-initiated, multicentre, multinational, and parallel-design RCT with one of the longest followup period at 24 months to evaluate the effect of biocompatible fluids on RRF. One hundred and eighty-five incident patients were randomised to receive neutral pH, lactate-buffered, low-GDP Balance fluid (Fresenius Medical Care, Bad Homburg, Germany; *n* = 93) or conventional, standard, lactate-buffered Stay-safe PD fluids (*n* = 92). Methodological quality as assessed by random sequence generation and allocation concealment was adequate. The primary outcome measure was the slope of RRF decline with secondary outcome measures, which comprised time to anuria, volume status, peritonitis-free survival, technique survival, patient survival, and adverse events. Although the rate of decline of renal function measured by the slopes of GFR did not reach statistical significance (−0.22 and −0.28 mL/min per 1.73 m^2^ per month (*P* = 0.17) in the first year and −0.09 and −0.10 mL/min per 1.73 m^2^ per month (*P* = 0.9) in the second year in the treatment and control groups, resp.), there was a significant delay in time to anuria (*P* = 0.009). There was no difference in volume status examined by body weight and blood pressure. Although the primary outcome did not reach statistical significance, it is important to acknowledge the importance of preservation of residual diuresis [49]. Findings from this trial are strengthened by the large sample size, involvement of patients from a range of centres and countries, with stratified randomisation strategy to minimise the centre effect on measured outcomes, and longer followup. Inclusion of incident patients who are dialysis naïve eliminates the possibility of bias introduced by different dialysis vintage. However, the study is limited by achieving lower than prespecified recruitment target (55% of target of 336 patients), absence of objective volume assessment (e.g., bioimpedance), and open-label design, which may have introduced cointervention bias. 

Eight other RCTs exclusively studied incident PD patients [30, 32, 33, 36, 37, 40, 45, 50, 51]. Of these, RRF benefit was reported in two trials [36, 37, 51, 52]. Although the study conducted by Kim and colleagues (*n* = 91) [36, 51, 52] was limited by a high dropout rate (24.2%), a trend towards improved preservation of residual GFR in the treatment group was demonstrated at 12 months (39.6 ± 50.2 versus 22.4 ± 18.6 L/week/1.73 m^2^, *P* = 0.057) and reached significance at 24 months (35.3 ± 6.86 versus 16.6 ± 4.36 L/week/1.73 m^2^, *P* = 0.011) [52]. There was a trend towards greater urine volume in the treatment group (750 ± 679 versus 532 ± 408 mL/day, *P* = 0.112) in the context of a significant reduction in daily peritoneal ultrafiltration (750 ± 350 versus 1047 ± 334 mL/day, *P* = 0.011) at the 12-month followup. Decrease in peritoneal ultrafiltration may have led to an increased urine output from hypervolaemia. However, this is less likely in the absence of significant difference in body weight, blood pressure, daily glucose loading, and the use of diuretics between the two groups. 

More recently, Lai and colleagues [37] reported the results of an open-label, multicentre, and parallel-design RCT involving 125 incident PD patients. Patients were assigned to either treatment (Gambrosol Trio, Gambro Lundia AB, Lund, Sweden (*n* = 41); Physioneal 40, Baxter Healthcare Corporation, Deerfield, IL, USA (*n* = 12); Balance (*n* = 5)) or control group (Dianeal PD-2, Baxter Healthcare Corporation (*n* = 43); ANDY-Disc, Fresenius Medical Care (*n* = 24)) for an average period of 3.6 years. Randomisation was instituted by the patient's training nursing officer at the individual renal centre, which raises concern for selection and allocation bias. Moreover, informed consent was obtained after the commencement of study at a median period of 30 months. In spite of using PD fluids with variable content of GDP (Table 2), the treatment group had higher urine output (745.7 ± 107.57 versus 475.1 ± 77.69 mL/day, *P* = 0.04) and slower median decline of both urine output (0.01 versus 0.33 mL/day, *P* = 0.004) and residual GFR (0.2 versus 0.56 L/min/1.73 m^2^/year, *P* = 0.05) at approximately 15 months. This study is limited by significant methodological flaws, and obtainment of informed consent after commencement of the trial is concerning. 

In contrast, lack of benefit in RRF with the use of biocompatible PD fluid was reported by Kim and colleagues [45] in their open-label, multi-centre, parallel-design RCT involving 26 incident PD patients over 12 months (2.3 ± 0.3 versus 1.8 ± 0.7 mL/min, *P* = NS). There was paucity in description of methodological process, including absence of clear reporting of randomisation technique, allocation concealment, and patient flow to assess for dropout rates. The study analysed the data from 26 patients, but 64 were initially recruited, and it was not possible to determine if these patients were randomised or even the reasons that led to their dropout. 

A recent open-label, multicentre, parallel-design RCT from Hong Kong [46] assessed the effect of NEPP regimen (two exchanges of Physioneal, one Nutrineal, and one exchange of Extraneal (Baxter); *n* = 77) against conventional PD fluids (Dianeal (Baxter); *n* = 73) in 150 incident CAPD patients. Although the study observed better preservation of daily urine volume in the treatment group (959 ± 515 versus 798 ± 615 mL/day, *P* = 0.02), they did not identify any significant difference in RRF (3.24 ± 1.98 versus 2.88 ± 2.43 mL/min/1.73 m^2^, *P* = NS) or the rate of decline in RRF (−0.76 ± 1.77 versus −0.91 ± 1.92 mL/min/1.73 m^2^/year, *P* = NS) at 12 months. Adequate randomisation technique and allocation concealment were adopted in this RCT. 

Inclusion of prevalent PD patients can cloud the interpretation of the outcome when the variable of interest is time dependent, such as RRF. A couple of RCTs included both incident and prevalent PD patients [34, 39], whereas only prevalent PD patients were involved in others [35, 38, 40–43, 47]. Of the three studies that showed benefit on RRF [34, 42, 43], the DIUREST study [34] was a parallel-design RCT conducted across three European countries with followup duration of 18 months (*n* = 80). Patients were centrally randomised to a treatment group to receive Gambrosol Trio (Gambro AB, Lund, Sweden) or conventional PD fluids from different manufacturers in single-compartment bags (Gambrosol for 50% of patients (Gambro AB), Stay-safe for 31% (Fresenius Medical Care, Bad Homburg, Germany) or Dianeal for 19% (Baxter GmbH, Unterschleißheim, Germany)). A significant benefit in preservation of monthly RRF change (−1.5%, 95% CI = −3.07%, +0.03% versus −4.3%, 95% CI = −6.8%, −2.06%, *P* = 0.0437) and urine volume (12 versus 38 mL/month, *P* = 0.0241) in the treatment group was reported; however, this should be interpreted cautiously in the context of inclusion of both incident and prevalent patients, unclear allocation concealment, high patient drop-out rate (51%), and use of per-protocol analysis. 

## 3. Peritoneal Solute Transport Rate 

Higher peritoneal solute transport rate (PSTR), assessed by the dialysate : plasma creatinine ratio (D : P Cr) from a peritoneal equilibration test (PET) [55], has been recognized as a significant risk factor for both mortality and technique failure in a number of large observational studies [56–60]. Although the exact mechanisms that lead to poor survival remain uncertain, rapid absorption of glucose with removal of osmotic gradient could contribute to impaired solute and fluid removal. Higher PSTR has been associated with greater appearance rate of interleukin-6 (IL-6) in PD effluent [61, 62], accumulation of advanced glycation end product (AGE), presence of GDP [63], and use of hypertonic glucose PD fluids [64]. This is biologically plausible, as a rise in vascularity followed by an increase in blood flow should result in greater PSTR. Intuitively, the use of biocompatible PD fluid has been postulated to slow the increase in PSTR. 

Nineteen RCTs have reported the effect of biocompatible PD fluid on PSTR [30–38, 41–45, 48, 51, 52, 65, 69]. The outcomes are conflicting, with a number of studies showing a decrease [35], an increase [37, 43, 44, 46, 51, 52], and no change [30, 32–34, 38, 41, 42, 45, 48, 65, 69]. In general, PSTR increases with time on PD, therefore, similar to RRF, inclusion of prevalent PD patients [31, 34, 35, 38, 41–43, 48, 65, 69] and crossover design [35, 38, 42, 43, 48, 65] creates a dilemma in the understanding of the outcome. Furthermore, interpretation of studies that showed greater PSTR in the treatment group should be done carefully as the difference was already present at the baseline (or month 1) in three studies [36, 37, 44, 51, 52, 70]. Kim and colleagues [36, 51, 52] reported a significant difference between treatment and control groups at baseline (0.72 ± 0.1 versus 0.67 ± 0.1, *P* = 0.001) and at 12 months (0.72 ± 0.11 versus 0.64 ± 0.08, *P* = 0.001). However, within-group analysis failed to show significant difference over the 12-month period. A large variation in PSTR between PD patients is well recognised [71]. Therefore, a difference at baseline may not be due to the biocompatible PD fluid, and the trend in PSTR over time may be of greater importance. 

The trend in PSTR was reported in the Euro-Balance Trial [43]. In this multicentre, open-label, crossover design RCT, 86 prevalent PD patients from 22 centres in 11 European countries were randomly allocated to conventional, acidic, lactate-buffered fluid (Stay-safe; Fresenius Medical Care, Bad Homburg, Germany) or neutral pH, lactate-buffered, low-GDP fluid (Balance; Fresenius Medical Care, Bad Homburg, Germany) for 12 weeks. There was no washout period between the two study periods. *Per-protocol* analysis was performed in 71 patients who completed the trial. Patients in the group I started receiving conventional fluids for the first 12 weeks followed by biocompatible fluid, and the order was reversed for patients in group II. In group I (*n* = 36), PSTR was higher whilst receiving biocompatible PD fluid (0.63 [0.34–0.89] versus 0.59 [0.35–0.80], *P* = 0.008) and similar outcome was reported in group II (0.60 [0.38–0.80] versus 0.56 [0.42–0.80], *P* = 0.0003). The decrease in PSTR with the use of biocompatible PD fluid has been reported by only one trial [35]. This study was a multicentre, open-label, crossover design RCT involving 28 prevalent patients. Following a 4-week run-in period, patients underwent two consecutive 12-week study periods, in randomised order, in which PD was performed with a neutral-pH PD fluid containing 34 mmol/L bicarbonate (BicaVera 170/180/190; Fresenius Medical Care, Bad Homburg, Germany) or a conventional PD fluid with 35 mmol/L lactate buffer (pH 5.5, CAPD 17/18/19; Fresenius Medical Care). The two treatment phases were separated by a 4-week washout period with a lactate-buffered PD fluid. *Per-protocol* analysis was performed in the twenty patients who completed both phases. A significant decrease in 4-hour D : P Cr during the treatment phase (0.67 ± 0.14 versus 0.70 ± 0.12, *P* < 0.05) was reported. Although these two trials were multinational and multicentre, they suffered from methodological problems including relatively small sample size, per protocol analysis, crossover design, and short followup duration. The latter two issues are particularly relevant given the time-dependent nature of PSTR and the risk of carryover effect of the PD fluids used. Therefore, the effect or lack of effect posed with the use of biocompatible PD fluid remains to be unknown. 

## 4. Peritoneal Ultrafiltration 

The decrease in peritoneal ultrafiltration (UF) is an important cause of technique failure [4]. Although it is largely driven by loss of osmotic gradient from higher PSTR with time on PD, disproportionate decrease in UF capacity can occur [72]. This is thought to result from an increase in membrane fibrosis, thereby compromising osmotic conductance independent of PSTR [73]. Severe fibrosis in the peritoneum from morphologic examination has been attributed as a consequence of continuous exposure to “unphysiologic” PD fluids [13]. However, accurate interpretation of the implication of UF volume as a clinical outcome is complex, as there are many variables that can affect its level, such as body's fluid status, urine volume, PSTR, glucose load, and the use of 7.5% icodextrin. 

Of the eighteen RCTs [30–38, 40–44, 48, 51, 52, 65–67] reporting UF, six studies showed a decrease in UF with the use of biocompatible PD fluids [36, 37, 43, 44, 51, 52, 67]. Interestingly, five RCTs within this category reported an increase in the urine volume with the use of biocompatible PD fluids [36, 37, 43, 44, 51, 52]. This highlights the importance of interpreting data in the context of other parameters present. 

An increase in UF with the use of biocompatible fluid was reported in only two RCTs [31, 41]. Both studies were performed in prevalent PD patients, and neither of the studies showed any difference in RRF between groups. Choi and colleagues [31] performed a single-centre, open-label, parallel-design RCT over 12 months. Of the 104 patients who were randomised, 66 patients were anuric at the time of enrolment with median PD duration of 67 months in the treatment group (*n* = 51) and 70.4 months in the control group (*n* = 53). Daily UF was significantly greater in the treatment group (1301.3 ± 597.6 versus 981.7 ± 538.8 mL/day, *P* < 0.05) in spite of similar glucose load (151.4 ± 54.5 versus 167.3 ± 38.8 g/day). Randomisation technique or allocation concealment were not clearly described, and the study suffered from a moderately high dropout rate (35%). 

Similarly, Tranaeus [41] conducted an open-label, parallel-design RCT across 17 European nephrology centres in 106 prevalent PD patients with mean baseline RRF of 2.8 mL/min/1.73 m^2^ over 12 months. Statistically significant difference (*P* < 0.05) in favour of biocompatible PD fluid was demonstrated (numerical data is not reported in the study). Stratified randomisation block technique was adopted; however, allocation concealment method was not clearly described. Less than half of the patients (*n* = 44) completed the study, which raises the possibility of attrition bias. Based on these two studies, perhaps the use of biocompatible PD fluid may be favoured to improve peritoneal UF in prevalent PD patients. However, these findings were not reproduced in other trials which included prevalent patients [35, 38, 42, 43, 48, 65–67]. Interestingly, all of those eight RCTs were crossover in design. 

## 5. Peritonitis 

The use of biocompatible PD fluids has been associated with reduction in peritonitis in an observational study [74]. This is supported by a number of *in vitro* and *ex vivo* studies that have demonstrated improvement in cellular function, in particular in the host immune system and an increase in markers of membrane integrity with their use [9, 10, 23, 24]. Peritonitis is an important cause of higher technique failure in Australia and New Zealand [4] and has been associated with greater mortality [5]. 

Disappointingly, however, of the 14 RCTs that reported peritonitis [30, 32–36, 38–44, 46, 47, 50–52, 68], only two showed significant benefit with the use of biocompatible PD fluids. The balANZ trial reported a significant delay in time to the first peritonitis episode (*P* = 0.01) and lower overall rates of peritonitis in the treatment group (0.30 versus 0.49 episodes per year, *P* = 0.01). Likewise, a significant reduction in peritonitis rate was demonstrated in the treatment group (1 : 51 patient-months versus 1 : 19 patient-months, *P* < 0.05) by Tranaeus [41]. No study has reported significant increase in peritonitis risk with the use of biocompatible PD fluids. 

Of the trials that showed no benefit, only the RCT conducted by Srivastava and colleagues [50] was powered adequately to examine the peritonitis. This was an extension study of an open-label, parallel-design, single-centre RCT (*n* = 118, dropout 21.7%) with initial followup of 12 months [32]. Enrolment into the study continued to achieve sufficient power to report any statistically significant difference in peritonitis episodes, which resulted in the inclusion of a large number of incident patients (*n* = 267). The treatment group received biocompatible PD solutions (either Physioneal or Balance) and control group received conventional PD solutions (either Dianeal or Stay-safe). The patients who used Baxter system (85% overall) were additionally allowed to use Extraneal or Nutrineal during the study duration. Patients were allowed to use different connectology (1-2 connections) that was felt to be best suited to each individual. There were 227 peritonitis episodes suffered by the patients, with an at-risk period of 7408 patient-months. Peritonitis rate for the treatment group was 1 : 34.7 versus 1 : 31.5 months in the control group (*P* = 0.61). Although this study was strengthened by large patient numbers, allowance of systems requiring different number of connections, thereby introducing variable risk of contamination and a variety of PD fluid types with varying contents (e.g., GDP, buffer system), could have introduced bias. 

## 6. Pain 

Inflow pain is generally attributed to the acidity (pH 5.2 to 5.5) of conventional lactate-buffered PD fluids. Although it is often temporary, it can be a troublesome complication in some PD patients to result in discontinuation of PD. Five RCTs assessed the effect of biocompatible PD fluids on inflow pain [14, 17, 41, 42, 47], with the majority of the studies reporting favourable result with the use of bicarbonate-buffered PD fluids. 

Mactier and colleagues [14] performed a double-blind, multicentre, multicountry, crossover design RCT in patients who had previously experienced inflow pain using conventional lactate-buffered PD fluids. Eighteen patients were recruited, and 17 completed the study protocol which comprised of two dialysis exchanges with each test solution determined by random allocation. Three visits were required to complete six exchanges in total (i.e., two exchanges per test solution). All tested fluids were of same glucose strength (3.86%), and pain was assessed by two methods (five-point verbal scale and the McGill Pain Questionnaire). Bicarbonate-buffered PD fluids were associated with significant reductions in inflow pain using both assessment methods. Bicarbonate/lactate-buffered PD fluid performed the best in terms of improving alleviating pain when all pain variables were assessed. However, there was a large variation within the eight participating centres in the frequency of inflow pain, which raises the concern for centre-related effects. 

Three other RCTs also reported significant benefit with the use of bicarbonate- or bicarbonate/lactate-buffered PD fluids [17, 41, 47]. Level of pain was measured using different tools devised during each trial in a form of questionnaire. For instance, Fusshoeller and colleagues conducted a single-centre, open-label, crossover design RCT in 14 prevalent PD patients [17]. Patients were randomised to have automated PD with either conventional fluid (Dianeal; Baxter Healthcare SA, County Mayo, Ireland) or a bicarbonate/lactate-based neutral fluid (Physioneal; Baxter Healthcare SA, County mayo, Ireland). After 6 months, both groups changed fluids. There was no washout period. Dialysate inflow pain was assessed with the use of a patient questionnaire conducted at baseline visit (1 = no pain; 5 = very intense) and at the end of the 5 months of treatment with each of the PD fluids. Similar findings were reported by Tranaeus [41]; there was a significant reduction in dialysate inflow pain in the treatment group (0.46 ± 0.93 versus 1.67 ± 0.70; *P* = 0.05). 

Feriani and colleagues [47] conducted a multicentre, open-label, parallel-design RCT over a 24-week period in prevalent PD patients (*n* = 123). Patients were randomly allocated to receive either a bicarbonate- or lactate-buffered PD fluid. Adverse symptoms were recorded using a standardized questionnaire (higher score indicating increase in severity) assessing local (pain during infusion, constipation, and diarrhoea), uraemic (itching, headache, restless legs, tiredness, and loss of appetite), and volume (thirst, ankle swelling, abdominal fullness, difficulty in maintaining correct weight, circulatory troubles, and shortness of breath) effects. Significant improvement in “local effects” was shown in the treatment group (0.25 ± 0.60 versus 0.45 ± 0.87, *P* < 0.01). The results from these three RCTs should be interpreted with caution as they were open-label RCTs leading to possible performance bias. 

A multicentre, open-label, cross-over design RCT conducted across three European countries including 53 prevalent PD patients was conducted by Weiss and colleagues [42]. Following a 2-week run-in phase, patients were randomised to receive either standard lactate-buffered PD fluids or purely bicarbonate-buffered PD fluids (Fresenius Medical Care, Bad Homburg, Germany) for 12 weeks, following which the treatment fluids were switched and continued for further 12 weeks. After completing this phase, pain assessment was performed under blinded administration condition of four exchanges in a randomised order. Twenty-seven patients who completed both treatment phases were included for analyses, and twenty-three proceeded to pain assessment. Pain intensity was assessed using McGill Pain Questionnaire, with similar outcomes between the two groups. In specific, 4 of 23 patients reported pain with both solutions during inflow. 

## 7. Conclusion 

There has been an increase in a number of published RCTs that compare the clinical outcome from the use of biocompatible PD fluids over the past decade. The results are generally in favour of or at least neutral with regards to RRF, PD peritonitis, and inflow pain in those who received biocompatible PD fluids. Its impact on peritoneal membrane function (i.e., PSTR and UF) remains uncertain. Some of the variability in the reported outcomes stem from flaws in study design, inclusion of patients from different dialysis vintage, inadequate statistical power to assess hard endpoints (e.g., mortality, technique failure), high dropout rates, and adoption of inappropriate analytical methods. Predominant use of open-label designs introduce cointervention and observer biases. Meta-analysis of all RCTs to clarify whether the use of biocompatible fluids translates into important clinical benefits is currently in progress [75]. The outcome of the analyses may provide further evidence for or against the use of these products. In the future, a large RCT with adequate statistical power to assess hard endpoints such as patient and technique survivals with the use of biocompatible PD fluids is needed. 

## Figures and Tables

**Table 1:** Adverse effects mediated by conventional peritoneal dialysis fluids.

Characteristics of fluid	Adverse effects
Acidic pH (5.0–5.8)	Pain [14, 17]
	Compromised mesothelial cell viability [8, 18]
Lactate buffer (30–40 mmol/L)	Compromised host-cell defense [11]
↑ Glucose concentrations (75.5 to 214 mmol/L)	Peritoneal membrane dysfunction [12]
	Vasculopathy via AGE [12]
	Compromised host-cell defense [6, 19]
↑ Glucose degradation product	Nephrotoxicity [15]
	Peritoneal membrane dysfunction [20, 21]

**Table 2:** Selected peritoneal dialysis fluids currently available in Australia.

Solution (manufacturer)	pH	Chambers	Buffer	Glucose degradation products (3-desoxyglycosone) [20, 53, 54]
Conventional PD fluids				

Dianeal (Baxter)	5.2	Single	Lactate (35–40 mmol/L)	↑↑↑ (525 *μ*mol/L)
Stay-safe (Fresenius)	5.5	Single	Lactate (40 mmol/L)	↑↑ (172–324 *μ*mol/L)

Biocompatible PD fluids				

Physioneal (Baxter)	7.4	Double	Lactate (10–15 mmol/L)/bicarbonate (25 mmol/L)	↓ (253 *μ*mol/L)
Balance (Fresenius)	7.0	Double	Lactate (35 mmol/L)	↓↓ (42 *μ*mol/L)
BicaVera (Fresenius)	7.4	Double	Bicarbonate (34/39 mmol/L)	↓↓ (42 *μ*mol/L)
Gambrosol Trio (Fresenius)	6.5	Triple	Lactate (39–41 mmol/L)	↓↓ (65 *μ*mol/L)

**Table 3:** Characteristics of populations and interventions in the randomised controlled trials assessing clinical outcomes with the use of biocompatible fluids in PD.

Study ID [reference]	Interventions	No. of patients	Study design	Outcome				Followup
		(incident/prevalent)	(parallel/crossover) (no. of centres)	RRF	PSTR	UF	Peritonitis
Neutral pH, lactate-buffered, low-GDP versus acidic pH, lactate-buffered, high-GDP fluids								

Bajo et al. [30]	Balance versus Stay-safe	33 (I)	Parallel (2)	—	—	—	—	24 months
Johnson et al. [44]	Balance versus Stay-safe	185 (I)	Parallel (16)	↑	↑	↓	↓	24 months
Kim et al. [36]	Balance versus Stay-safe	91 (I)	Parallel (4)	↑	↑	↓	—	12 months
Kim et al. [45]	Balance versus Stay-safe	26 (I)	Parallel (2)	—	—	NA	NA	12 months
Szeto et al. [40]	Balance versus Stay-safe	50 (I)	Parallel (1)	—	NA	—	—	12 months
Williams et al. [43]	Balance versus Stay-safe	86 (P)	Crossover (22)	↑	↑	↓	—	24 weeks
Choi et al. [31]	Balance versus Conventional (Dianeal, Stay-safe)	104 (P)	Parallel (1)	—	—	↑	NA	12 months
Haag-Weber et al. [34]	Gambrosol Trio versus Gambrosol	69 (I + P)	Parallel (5)	↑	—	—	—	18 months
Rippe et al. [39]	Gambrosol Trio versus Gambrosol	21 (I + P)	Parallel (9)	—	—	NA	—	24 months
Lai et al. [37]	Biocompatible (Balance, Gambrosol Trio, Physioneal) versus Conventional (Dianeal, ANDY-Disc)	125 (I)	Parallel (4)	↑	↑	↓	NA	2.3 years
Fan et al. [32]	Biocompatible (Balance or Physioneal) versus Conventional (Stay-safe or Dianeal)	118 (I)	Parallel (1)	—	—	—	—	12 months
Srivastava et al. [50]	Biocompatible (Balance or Physioneal) versus Conventional (Stay-safe or Dianeal)	267 (I)	Parallel (1)	NA	NA	NA	—	~2.3 years

Neutral pH, bicarbonate (±lactate)-buffered, low-GDP versus acidic pH, lactate-buffered, high-GDP fluids								

Fischback et al. [65]	Physioneal (25 mmol/L bicarbonate/15 mmol/L lactate) versus Dianeal	6 (P)	Cross-over (2)	NA	—	—	NA	2 days
Fusshoeller et al. [17]	Physioneal (25 mmol/L bicarbonate/15 mmol/L lactate) versus Dianeal	14 (P)	Cross-over (1)	NA	NA	NA	NA	12 months
John et al. [66]	Physioneal (25 mmol/L bicarbonate/15 mmol/L lactate) versus Dianeal	10 (P)	Cross-over (1)	NA	NA	—	NA	2 days
Pajek et al. [38]	Physioneal (25 mmol/L bicarbonate/15 mmol/L lactate) versus Dianeal	21 (P)	Cross-over (1)	—	—	—	NA	6 months
Parikova et al. [48]	Physioneal (25 mmol/L bicarbonate/15 mmol/L lactate) versus Dianeal	10 (P)	Cross-over (1)	NA	—	—	NA	2 days
Tranaeus [41]	25 mmol/L bicarbonate/15 mmol/L lactate PD fluid versus 40 mmol/L lactate-buffered PD fluid	106 (P)	Parallel (17)	—	—	↑	↓	12 months
Fang et al. [67]	Physioneal (25 mmol/L bicarbonate/15 mmol/L lactate) versus Dianeal	18 (P)	Cross-over (1)	NA	NA	↓	NA	2 days
Mactier et al. [14]	Bicarbonate/lactate (25/15 mmol/L) versus bicarbonate (38 mmol/L) versus lactate (40 mmol/L)	18 (P)	Cross-over (8)	NA	NA	NA	NA	3 days
Coles et al. [68]	Bicarbonate/lactate (25/15 mmol/L) versus bicarbonate (38 mmol/L) versus lactate (40 mmol/L)	59 (P)	Parallel (5)	NA	—	NA	—	2 months
Feriani et al. [47]	Bicavera (bicarbonate 34 mmol/L) versus lactate (35 mmol/L)	123 (P)	Parallel (14)	—	NA	NA	—	24 weeks
Haas et al. [35]	Bicavera (bicarbonate 34 mmol/L) versus lactate (35 mmol/L)	28 (P)	Cross-over (6)	—	↓	—	—	6 months
Fernandez-Perpén et al. [33]	Bicavera (bicarbonate 34 mmol/L) versus lactate (35 mmol/L)	31 (I)	Parallel (2)	—	—	—	—	24 months
Weiss et al. [42]	Bicarbonate (bicarbonate 34 mmol/L) versus lactate (35 mmol/L)	53 (P)	Cross-over (13)	↑	—	—	NA	6 months

Glucose sparing (NEPP) versus acidic pH, lactate-buffered, high-GDP fluids								

Lui [46]	Physioneal (25 mmol/L bicarbonate/15 mmol/L lactate), Extraneal (7.5% icodextrin), Nutrineal (1.1% amino acid) versus Dianeal	150 (I)	Parallel (8)	—	↑	—	—	

RRF: residual renal function; PSTR: peritoneal solute transport rate; UF: ultrafiltration; NEPP: Nutrineal, Extraneal, Physioneal, and physioneal; ↓: decrease, ↑: increase; —: no change; N/A: not available.
